# A second ortho­rhom­bic polymorph of (*Z*)-3-(9-anthr­yl)-1-(2-thien­yl)prop-2-en-1-one^1^

**DOI:** 10.1107/S1600536810000061

**Published:** 2010-01-09

**Authors:** Suchada Chantrapromma, Thitipone Suwunwong, Nawong Boonnak, Hoong-Kun Fun

**Affiliations:** a Crystal Materials Research Unit, Department of Chemistry, Faculty of Science, Prince of Songkla University, Hat-Yai, Songkhla 90112, Thailand; b X-ray Crystallography Unit, School of Physics, Universiti Sains Malaysia, 11800 USM, Penang, Malaysia

## Abstract

The title heteroaryl chalcone, C_21_H_14_OS, is a second ortho­rhom­bic polymorph which crystallizes in the space group *P*2_1_2_1_2_1_. The structure was previously reported [Fun *et al.* (2009 ▶). *Acta Cryst*. E**65**, o2168-o2169] in the space group *Pna*2_1_. The bond distances and angles are similar in both structures. In contrast, the overall crystal packing is different from that in the first ortho­rhom­bic *Pna*2_1_ polymorph in which mol­ecules were stacked into columns along the *b* axis and the thio­phene units of two adjacent columns were stacked in a head to tail fashion. In the present polymorph, mol­ecules are found to dimerize through a weak S⋯S inter­action [3.6513 (7) Å] and these dimers are arranged into sheets parallel to the *bc* plane. There are no classical hydrogen bonds in the packing which features short C⋯O [3.2832 (2)–3.6251 (9) Å], C⋯S [3.4879 (17)–3.6251 (19) Å] and S⋯O [2.9948 (16) Å] contacts, together with C—H⋯π inter­actions. Similar contacts were found in the other polymorph.

## Related literature

For bond-length data, see: Allen *et al.* (1987 ▶). For the structure of the first polymorph, see: Fun *et al.* (2009 ▶). For background to and applications of chalcones, see: Chantrapromma *et al.* (2009 ▶); Patil & Dharmaprakash (2008 ▶); Saydam *et al.* (2003 ▶); Suwunwong *et al.* (2009 ▶); Svetlichny *et al.* (2007 ▶). For the stability of the temperature controller used in the data collection, see Cosier & Glazer, (1986 ▶).
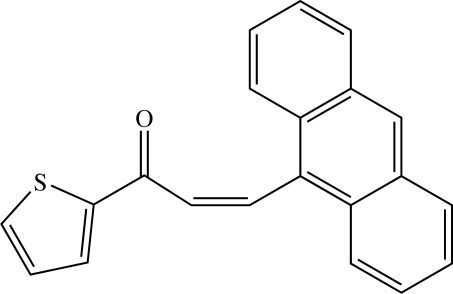


## Experimental

### 

#### Crystal data

C_21_H_14_OS
*M* *_r_* = 314.38
Orthorhombic, 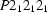

*a* = 5.5116 (1) Å
*b* = 14.8497 (2) Å
*c* = 18.3625 (3) Å
*V* = 1502.89 (4) Å^3^
*Z* = 4
Mo *K*α radiation
μ = 0.22 mm^−1^
*T* = 100 K
0.50 × 0.19 × 0.11 mm

#### Data collection

Bruker APEXII CCD area-detector diffractometer
Absorption correction: multi-scan (*SADABS*; Bruker, 2005 ▶) *T*_min_ = 0.900, *T*_max_ = 0.977
14062 measured reflections
4354 independent reflections
4035 reflections with *I* > 2σ(*I*)
*R*_int_ = 0.025

#### Refinement

*R*[*F* ^2^ > 2σ(*F* ^2^)] = 0.042
*wR*(*F* ^2^) = 0.108
*S* = 1.05
4354 reflections
254 parameters
H atoms treated by a mixture of independent and constrained refinement
Δρ_max_ = 0.79 e Å^−3^
Δρ_min_ = −0.62 e Å^−3^
Absolute structure: Flack (1983 ▶), 1830 Friedel pairs
Flack parameter: 0.04 (8)

### 

Data collection: *APEX2* (Bruker, 2005 ▶); cell refinement: *SAINT* (Bruker, 2005 ▶); data reduction: *SAINT*; program(s) used to solve structure: *SHELXTL* (Sheldrick, 2008 ▶); program(s) used to refine structure: *SHELXTL*; molecular graphics: *SHELXTL* software used to prepare material for publication: *SHELXTL* and *PLATON* (Spek, 2009 ▶).

## Supplementary Material

Crystal structure: contains datablocks global, I. DOI: 10.1107/S1600536810000061/sj2716sup1.cif

Structure factors: contains datablocks I. DOI: 10.1107/S1600536810000061/sj2716Isup2.hkl

Additional supplementary materials:  crystallographic information; 3D view; checkCIF report

## Figures and Tables

**Table 1:** Hydrogen-bond geometry (Å, °) *Cg*_1_, *Cg*_2_ and *Cg*_3_ are the centroids of the S1/C18–C21, C1–C6 and C8–C13 rings, respectively.

*D*—H⋯*A*	*D*—H	H⋯*A*	*D*⋯*A*	*D*—H⋯*A*
C5—H5*A*⋯*Cg*1^i^	0.91 (3)	2.64 (3)	3.443 (2)	149 (2)
C15—H15*A*⋯*Cg*2^ii^	0.95 (2)	2.74 (2)	3.565 (2)	146.4 (17)
C21—H21*A*⋯*Cg*3^iii^	1.04 (3)	2.91 (3)	3.711 (2)	134.4 (19)

Symmetry codes: (i) 
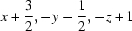
; (ii) 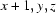; (iii) 
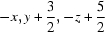
.

**Fig. 1.**
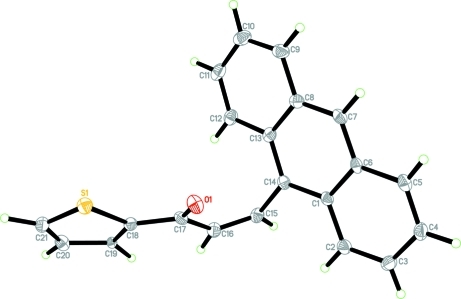
The molecular structure of the title compound, showing 50% probability displacement ellipsoids and the atom-numbering scheme.

**Fig. 2.**
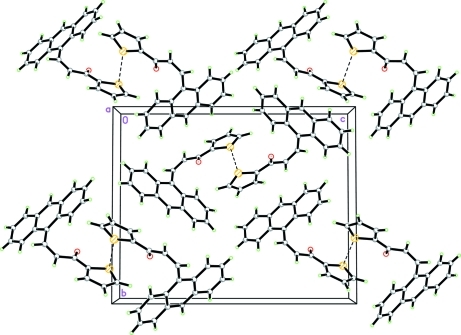
The crystal packing of the title compound viewed along the a axis. S···S contacts are shown as dashed lines.

## supplementary crystallographic information

### Comment

In continuation of our study of chalcone derivatives (Chantrapromma *et al.*, 2009; Fun *et al.*, 2009; Suwunwong *et al.*, 2009) which can be used for non-linear optical (NLO) materials (Patil & Dharmaprakash, 2008), fluorescent materials (Svetlichny *et al.*, 2007) and bioactive compounds (Saydam *et al.*, 2003), the title heteroaryl chalcone (I) was synthesized and its crystal structure was previously reported in the orthorhombic space group *Pna*2_1_ (Fun *et al.*, 2009). In the present work, the compound crystallized in the orthorhombic space group *P*2_1_2_1_2_1_ from an ethanol/acetone (1:1) solvent mixture, while the crystal of the *Pna*2_1_ form crystallized from hot ethanol. (I) exhibits fluorescence with the maximum emission at 402 nm when the compound is excited at 335 nm.

The molecule of (I) (Fig. 1) exists in an *Z* configuration with respect to the C15═C16 double bond and the torsion angle C14–C15–C16–C17 = -2.9 (3)° [compared to -3.7 (7)° in one molecule and -4.0 (7)° in the other in the *Pna*2_1_ polymorph which contains two molecules in an asymmetric unit]. The molecule of the present polymorph is less twisted as indicated by the interplanar angles between thiophene and anthracene rings being 56.36 (7)° and the least squares plane through the prop-2-en-1-one unit (C15–C17/O1) makes interplanar angles of 12.2 and 68.00 (11)° with the thiophene and anthracene rings, respectively [the corresponding values are 75.07 (17), 13.1 (3) and 71.2 (3)° in one molecule and 76.32 (17), 15.2 (3) and 72.3 (3)° in the other for the *Pna*2_1_ polymorph]. Bond distances are within normal ranges (Allen *et al.*, 1987).

In the crystal packing (Fig. 2), molecules are found to dimerize through a non-bonding S···S interaction [S···S = 3.6513 (7) Å]. The dimers are arranged into sheets parallel to the *bc* plane. These sheets are stacked along the *a* axis. The intermolecular interactions and short contacts are almost similar in both polymorph. There is no classic hydrogen bond and the crystal is consolidated by short C···O [3.2832 (2)–3.6251 (9) Å], C···S [3.4879 (17)–3.6251 (19) Å] and S···O [2.9948 (16) Å] contacts, as well as C—H···π interactions (Table 1); *Cg*_1_, *Cg*_2_ and *Cg*_3_ are the centroids of the S1/C18–C21, C1–C6 and C8–C13 rings, respectively.

### Experimental

The title compound was synthesized as reported by Fun *et al.* (2009). Yellow block-shaped single crystals of the title compound suitable for *x*-ray structure determination were recrystallized from ethanol/acetone (1:1 *v*/*v*) by slow evaporation of the solvent at room temperature over several days, Mp. 400–401 K.

### Refinement

The H atom attached to C19 was placed in a calculated position, with d(C—H) = 0.93 Å, *U*_iso_ = 1.2*U*_eq_(C). The remaining H atoms were located from the difference maps and refined isotropically. The highest residual electron density peak is located at 0.04 Å from C19 and the deepest hole is 0.07 Å from C20. A total of 1830 Friedel pairs were used to determine the absolute configuration.

### Crystal data

**Table:**

C_21_H_14_OS	*D*_x_ = 1.390 Mg m^−^^3^
*M**_r_* = 314.38	Melting point = 400–401 K
Orthorhombic, *P*2_1_2_1_2_1_	Mo *K*α radiation, λ = 0.71073 Å
Hall symbol: P 2ac 2ab	Cell parameters from 4354 reflections
*a* = 5.5116 (1) Å	θ = 1.8–30.0°
*b* = 14.8497 (2) Å	µ = 0.22 mm^−^^1^
*c* = 18.3625 (3) Å	*T* = 100 K
*V* = 1502.89 (4) Å^3^	Block, yellow
*Z* = 4	0.50 × 0.19 × 0.11 mm
*F*(000) = 656	

### Data collection

**Table:**

Bruker APEXII CCD area-detector diffractometer	4354 independent reflections
Radiation source: sealed tube	4035 reflections with *I* > 2σ(*I*)
graphite	*R*_int_ = 0.025
φ and ω scans	θ_max_ = 30.0°, θ_min_ = 1.8°
Absorption correction: multi-scan (*SADABS*; Bruker, 2005)	*h* = −7→5
*T*_min_ = 0.900, *T*_max_ = 0.977	*k* = −17→20
14062 measured reflections	*l* = −25→18

### Refinement

**Table:**

Refinement on *F*^2^	Secondary atom site location: difference Fourier map
Least-squares matrix: full	Hydrogen site location: inferred from neighbouring sites
*R*[*F*^2^ > 2σ(*F*^2^)] = 0.042	H atoms treated by a mixture of independent and constrained refinement
*wR*(*F*^2^) = 0.108	*w* = 1/[σ^2^(*F*_o_^2^) + (0.0502*P*)^2^ + 0.8977*P*] where *P* = (*F*_o_^2^ + 2*F*_c_^2^)/3
*S* = 1.05	(Δ/σ)_max_ = 0.001
4354 reflections	Δρ_max_ = 0.79 e Å^−^^3^
254 parameters	Δρ_min_ = −0.62 e Å^−^^3^
0 restraints	Absolute structure: Flack (1983), 1830 Friedel pairs
Primary atom site location: structure-invariant direct methods	Flack parameter: 0.04 (8)

### Special details

**Table:**

Experimental. The crystal was placed in the cold stream of an Oxford Cryosystems Cobra open-flow nitrogen cryostat (Cosier & Glazer, 1986) operating at 120.0 (1) K.
Geometry. All e.s.d.'s (except the e.s.d. in the dihedral angle between two l.s. planes) are estimated using the full covariance matrix. The cell e.s.d.'s are taken into account individually in the estimation of e.s.d.'s in distances, angles and torsion angles; correlations between e.s.d.'s in cell parameters are only used when they are defined by crystal symmetry. An approximate (isotropic) treatment of cell e.s.d.'s is used for estimating e.s.d.'s involving l.s. planes.
Refinement. Refinement of *F*^2^ against ALL reflections. The weighted *R*-factor *wR* and goodness of fit *S* are based on *F*^2^, conventional *R*-factors *R* are based on *F*, with *F* set to zero for negative *F*^2^. The threshold expression of *F*^2^ > σ(*F*^2^) is used only for calculating *R*-factors(gt) *etc*. and is not relevant to the choice of reflections for refinement. *R*-factors based on *F*^2^ are statistically about twice as large as those based on *F*, and *R*- factors based on ALL data will be even larger.

### Fractional atomic coordinates and isotropic or equivalent isotropic displacement parameters (Å^2^)

**Table:**

	*x*	*y*	*z*	*U*_iso_*/*U*_eq_	
S1	0.79023 (9)	0.82740 (3)	0.98167 (3)	0.01920 (11)	
O1	0.6379 (3)	0.74101 (10)	0.84157 (8)	0.0208 (3)	
C1	0.6215 (3)	0.62511 (12)	0.66251 (10)	0.0142 (3)	
C2	0.6199 (4)	0.69201 (13)	0.60656 (10)	0.0176 (4)	
H2A	0.747 (4)	0.7404 (15)	0.6071 (12)	0.019 (6)*	
C3	0.4549 (4)	0.68828 (13)	0.55096 (10)	0.0193 (4)	
H3A	0.459 (4)	0.7304 (16)	0.5132 (14)	0.022 (6)*	
C4	0.2761 (4)	0.61930 (13)	0.54827 (11)	0.0198 (4)	
H4A	0.150 (5)	0.6169 (17)	0.5090 (13)	0.026 (6)*	
C5	0.2685 (4)	0.55527 (13)	0.60124 (10)	0.0184 (4)	
H5A	0.155 (5)	0.5111 (18)	0.6021 (14)	0.033 (7)*	
C6	0.4394 (3)	0.55565 (12)	0.65992 (10)	0.0146 (3)	
C7	0.4332 (4)	0.49043 (12)	0.71450 (10)	0.0169 (4)	
H7A	0.311 (4)	0.4456 (14)	0.7129 (11)	0.010 (5)*	
C8	0.6064 (4)	0.48855 (12)	0.76949 (10)	0.0159 (4)	
C9	0.6059 (4)	0.41897 (13)	0.82390 (11)	0.0213 (4)	
H9A	0.479 (4)	0.3744 (16)	0.8218 (13)	0.019 (6)*	
C10	0.7811 (4)	0.41528 (14)	0.87607 (11)	0.0240 (4)	
H10A	0.788 (5)	0.3656 (16)	0.9099 (13)	0.022 (6)*	
C11	0.9682 (4)	0.48106 (15)	0.87753 (11)	0.0222 (4)	
H11A	1.103 (5)	0.4744 (16)	0.9123 (13)	0.023 (6)*	
C12	0.9731 (4)	0.54951 (14)	0.82813 (10)	0.0189 (4)	
H12A	1.091 (5)	0.5966 (17)	0.8299 (13)	0.027 (7)*	
C13	0.7917 (4)	0.55659 (12)	0.77254 (9)	0.0156 (3)	
C14	0.7917 (3)	0.62627 (12)	0.72009 (9)	0.0143 (3)	
C15	0.9746 (4)	0.69969 (13)	0.72208 (10)	0.0172 (4)	
H15A	1.077 (4)	0.7024 (15)	0.6807 (12)	0.016 (6)*	
C16	1.0028 (4)	0.76158 (13)	0.77411 (10)	0.0178 (4)	
H16A	1.138 (5)	0.8035 (17)	0.7711 (13)	0.026 (6)*	
C17	0.8452 (3)	0.77098 (12)	0.83955 (10)	0.0153 (3)	
C18	0.9491 (3)	0.82083 (12)	0.90132 (9)	0.0146 (3)	
C19	1.1727 (3)	0.86783 (12)	0.90518 (10)	0.0146 (2)	
H19A	1.2863	0.8714	0.8678	0.018*	
C20	1.1971 (3)	0.90888 (11)	0.97520 (10)	0.0146 (2)	
H20A	1.351 (5)	0.9445 (17)	0.9876 (14)	0.031 (7)*	
C21	1.0083 (4)	0.89174 (13)	1.02086 (11)	0.0198 (4)	
H21A	0.993 (5)	0.9095 (18)	1.0753 (15)	0.034 (7)*	

### Atomic displacement parameters (Å^2^)

**Table:**

	*U*^11^	*U*^22^	*U*^33^	*U*^12^	*U*^13^	*U*^23^
S1	0.0185 (2)	0.0204 (2)	0.0188 (2)	−0.00094 (17)	0.00214 (18)	−0.00059 (18)
O1	0.0163 (6)	0.0219 (7)	0.0243 (7)	−0.0038 (6)	0.0037 (6)	−0.0053 (6)
C1	0.0159 (8)	0.0132 (8)	0.0136 (8)	0.0006 (7)	0.0018 (6)	−0.0028 (6)
C2	0.0200 (9)	0.0167 (8)	0.0162 (8)	0.0000 (7)	0.0013 (7)	−0.0014 (7)
C3	0.0234 (9)	0.0181 (9)	0.0163 (8)	0.0033 (7)	−0.0002 (7)	0.0009 (7)
C4	0.0211 (9)	0.0210 (9)	0.0174 (8)	0.0038 (8)	−0.0038 (8)	−0.0049 (7)
C5	0.0182 (9)	0.0167 (8)	0.0204 (8)	0.0001 (7)	−0.0007 (8)	−0.0057 (7)
C6	0.0140 (8)	0.0143 (8)	0.0155 (8)	0.0007 (7)	−0.0001 (7)	−0.0056 (7)
C7	0.0187 (9)	0.0131 (8)	0.0188 (8)	−0.0010 (7)	0.0030 (7)	−0.0038 (7)
C8	0.0192 (9)	0.0142 (8)	0.0143 (8)	0.0023 (7)	0.0050 (7)	−0.0007 (7)
C9	0.0279 (11)	0.0152 (9)	0.0208 (9)	0.0012 (8)	0.0065 (8)	0.0002 (7)
C10	0.0325 (11)	0.0215 (9)	0.0179 (8)	0.0078 (9)	0.0061 (9)	0.0032 (7)
C11	0.0254 (10)	0.0262 (10)	0.0149 (8)	0.0088 (9)	0.0012 (8)	0.0007 (8)
C12	0.0182 (9)	0.0219 (9)	0.0166 (8)	0.0029 (8)	0.0016 (7)	−0.0013 (7)
C13	0.0164 (8)	0.0170 (8)	0.0133 (7)	0.0038 (7)	0.0036 (7)	−0.0019 (6)
C14	0.0146 (7)	0.0144 (8)	0.0141 (7)	0.0014 (7)	0.0019 (7)	−0.0028 (6)
C15	0.0163 (8)	0.0213 (9)	0.0141 (8)	−0.0014 (7)	0.0026 (7)	0.0002 (7)
C16	0.0170 (9)	0.0198 (9)	0.0165 (8)	−0.0033 (7)	0.0013 (7)	0.0000 (7)
C17	0.0173 (9)	0.0118 (8)	0.0167 (8)	−0.0001 (7)	0.0011 (7)	−0.0010 (6)
C18	0.0161 (8)	0.0151 (8)	0.0125 (7)	0.0021 (7)	0.0022 (6)	0.0003 (7)
C19	0.0125 (5)	0.0149 (5)	0.0164 (6)	0.0005 (5)	−0.0024 (5)	−0.0019 (5)
C20	0.0125 (5)	0.0149 (5)	0.0164 (6)	0.0005 (5)	−0.0024 (5)	−0.0019 (5)
C21	0.0243 (9)	0.0190 (8)	0.0161 (8)	0.0040 (7)	−0.0012 (8)	−0.0017 (7)

### Geometric parameters (Å, °)

**Table:**

S1—C21	1.696 (2)	C9—H9A	0.96 (3)
S1—C18	1.7184 (18)	C10—C11	1.421 (3)
O1—C17	1.227 (2)	C10—H10A	0.97 (2)
C1—C14	1.414 (3)	C11—C12	1.363 (3)
C1—C2	1.429 (3)	C11—H11A	0.98 (3)
C1—C6	1.440 (3)	C12—C13	1.433 (3)
C2—C3	1.368 (3)	C12—H12A	0.96 (3)
C2—H2A	1.00 (2)	C13—C14	1.414 (2)
C3—C4	1.422 (3)	C14—C15	1.485 (3)
C3—H3A	0.93 (2)	C15—C16	1.335 (3)
C4—C5	1.361 (3)	C15—H15A	0.95 (2)
C4—H4A	1.00 (3)	C16—C17	1.489 (3)
C5—C6	1.431 (3)	C16—H16A	0.97 (3)
C5—H5A	0.91 (3)	C17—C18	1.470 (3)
C6—C7	1.394 (3)	C18—C19	1.418 (3)
C7—C8	1.390 (3)	C19—C20	1.429 (2)
C7—H7A	0.95 (2)	C19—H19A	0.9300
C8—C9	1.437 (3)	C20—C21	1.360 (3)
C8—C13	1.438 (3)	C20—H20A	1.02 (3)
C9—C10	1.361 (3)	C21—H21A	1.04 (3)
			
C21—S1—C18	92.02 (10)	C12—C11—H11A	119.5 (15)
C14—C1—C2	122.21 (17)	C10—C11—H11A	119.4 (14)
C14—C1—C6	119.73 (17)	C11—C12—C13	121.0 (2)
C2—C1—C6	118.05 (17)	C11—C12—H12A	122.4 (16)
C3—C2—C1	120.81 (18)	C13—C12—H12A	116.5 (16)
C3—C2—H2A	120.0 (13)	C14—C13—C12	122.62 (18)
C1—C2—H2A	119.1 (13)	C14—C13—C8	119.20 (17)
C2—C3—C4	121.04 (18)	C12—C13—C8	118.16 (17)
C2—C3—H3A	120.8 (15)	C13—C14—C1	120.04 (17)
C4—C3—H3A	118.2 (15)	C13—C14—C15	121.37 (16)
C5—C4—C3	119.99 (18)	C1—C14—C15	118.55 (16)
C5—C4—H4A	118.0 (15)	C16—C15—C14	127.01 (17)
C3—C4—H4A	122.0 (15)	C16—C15—H15A	118.3 (14)
C4—C5—C6	120.99 (19)	C14—C15—H15A	114.7 (14)
C4—C5—H5A	122.6 (17)	C15—C16—C17	125.05 (17)
C6—C5—H5A	116.4 (17)	C15—C16—H16A	119.3 (15)
C7—C6—C5	121.47 (17)	C17—C16—H16A	115.6 (15)
C7—C6—C1	119.43 (17)	O1—C17—C18	121.46 (17)
C5—C6—C1	119.10 (17)	O1—C17—C16	122.25 (17)
C8—C7—C6	121.27 (17)	C18—C17—C16	116.26 (16)
C8—C7—H7A	119.8 (13)	C19—C18—C17	128.65 (16)
C6—C7—H7A	118.9 (13)	C19—C18—S1	111.83 (13)
C7—C8—C9	121.21 (18)	C17—C18—S1	119.51 (14)
C7—C8—C13	120.14 (17)	C18—C19—C20	109.67 (16)
C9—C8—C13	118.65 (18)	C18—C19—H19A	125.2
C10—C9—C8	121.1 (2)	C20—C19—H19A	125.2
C10—C9—H9A	121.0 (14)	C21—C20—C19	113.75 (16)
C8—C9—H9A	117.8 (14)	C21—C20—H20A	126.4 (15)
C9—C10—C11	120.04 (18)	C19—C20—H20A	119.8 (15)
C9—C10—H10A	120.8 (16)	C20—C21—S1	112.71 (15)
C11—C10—H10A	119.0 (16)	C20—C21—H21A	127.5 (16)
C12—C11—C10	121.0 (2)	S1—C21—H21A	119.7 (16)
			
C14—C1—C2—C3	179.06 (17)	C9—C8—C13—C12	2.5 (3)
C6—C1—C2—C3	−2.1 (3)	C12—C13—C14—C1	174.11 (17)
C1—C2—C3—C4	1.7 (3)	C8—C13—C14—C1	−4.4 (3)
C2—C3—C4—C5	−0.5 (3)	C12—C13—C14—C15	−3.3 (3)
C3—C4—C5—C6	−0.4 (3)	C8—C13—C14—C15	178.18 (16)
C4—C5—C6—C7	179.95 (18)	C2—C1—C14—C13	−177.53 (17)
C4—C5—C6—C1	0.0 (3)	C6—C1—C14—C13	3.6 (3)
C14—C1—C6—C7	0.2 (3)	C2—C1—C14—C15	0.0 (3)
C2—C1—C6—C7	−178.75 (17)	C6—C1—C14—C15	−178.90 (16)
C14—C1—C6—C5	−179.92 (16)	C13—C14—C15—C16	−64.2 (3)
C2—C1—C6—C5	1.2 (3)	C1—C14—C15—C16	118.4 (2)
C5—C6—C7—C8	176.94 (17)	C14—C15—C16—C17	−2.9 (3)
C1—C6—C7—C8	−3.1 (3)	C15—C16—C17—O1	−21.8 (3)
C6—C7—C8—C9	−177.26 (17)	C15—C16—C17—C18	160.02 (19)
C6—C7—C8—C13	2.3 (3)	O1—C17—C18—C19	−172.54 (18)
C7—C8—C9—C10	177.47 (19)	C16—C17—C18—C19	5.7 (3)
C13—C8—C9—C10	−2.1 (3)	O1—C17—C18—S1	6.9 (2)
C8—C9—C10—C11	0.2 (3)	C16—C17—C18—S1	−174.86 (13)
C9—C10—C11—C12	1.3 (3)	C21—S1—C18—C19	0.62 (15)
C10—C11—C12—C13	−0.9 (3)	C21—S1—C18—C17	−178.93 (15)
C11—C12—C13—C14	−179.56 (18)	C17—C18—C19—C20	178.21 (17)
C11—C12—C13—C8	−1.0 (3)	S1—C18—C19—C20	−1.30 (19)
C7—C8—C13—C14	1.5 (3)	C18—C19—C20—C21	1.5 (2)
C9—C8—C13—C14	−178.92 (17)	C19—C20—C21—S1	−1.1 (2)
C7—C8—C13—C12	−177.10 (17)	C18—S1—C21—C20	0.27 (15)

### Hydrogen-bond geometry (Å, °)

**Table:**

*Cg*_1_, *Cg*_2_ and *Cg*_3_ are the centroids of the S1/C18–C21, C1–C6 and C8–C13 rings, respectively.

**Table:**

*D*—H···*A*	*D*—H	H···*A*	*D*···*A*	*D*—H···*A*
C5—H5A···Cg1^i^	0.91 (3)	2.64 (3)	3.443 (2)	149 (2)
C15—H15A···Cg2^ii^	0.95 (2)	2.74 (2)	3.565 (2)	146.4 (17)
C21—H21A···Cg3^iii^	1.04 (3)	2.91 (3)	3.711 (2)	134.4 (19)

Symmetry codes: (i) *x*+3/2, −*y*−1/2, −*z*+1; (ii) *x*+1, *y*, *z*; (iii) −*x*, *y*+3/2, −*z*+5/2.
